# Supplementary material for: Sliding Dynamics of Ring Chains on Two Asymmetric/Symmetric Chains in a Simple Slide-Ring Gel
Source: Polymers (Basel). 2021 Dec 26;14(1):79. doi: 10.3390/polym14010079 (PMC8747720; doi:10.3390/polym14010079)
Supplement: Supplementary file 1 [file polymers-14-00079-s001.zip › polymers-1476557-supplementary.pdf]

Supplementary Information

Sliding dynamics of ring chains on two asymmetric/symmetric chains in a simple slide-ring gel

Jiaxin Wu, Fuchen Guo , Ke Li and Linxi Zhang

S1 Effects of axial chain length on sliding dynamics

We calculated the diffusion coefficient D of ring chains sliding along two symmetric fluctuating axial chains, in which we changed the axial chain length from N_{axial}=100 to N_{axial} =200 and kept the other parameters unchanged. The dependence of D on the distance d between two symmetric axial chains is shown in Figure S1. From Figure S1, we found that there also exists a maximum value for D^{max} located at d*=16, which is the same as the case of N_{axial}=100 for ring chains sliding along the symmetric axial chains. Therefore, the similar conclusion can be made for different axial chain lengths.

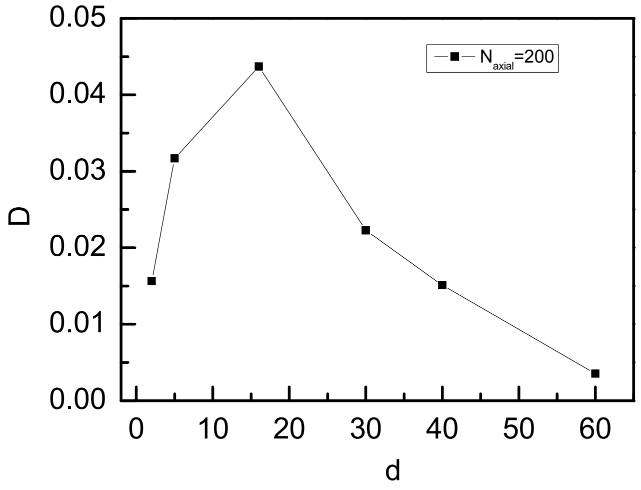

Figure S1. Diffusion coefficient D of ring chains sliding along two symmetric fluctuating axial chains (K_1=K_2=0) with different distances d between two symmetric fluctuating axial chains for N_{axial}=200.
